# Supplementary material for: Accuracy and Efficiency of Right-Lobe Graft Weight Estimation Using Deep-Learning-Assisted CT Volumetry for Living-Donor Liver Transplantation
Source: Diagnostics (Basel). 2022 Feb 25;12(3):590. doi: 10.3390/diagnostics12030590 (PMC8946991; doi:10.3390/diagnostics12030590)
Supplement: Supplementary file 1 [file diagnostics-12-00590-s001.zip › diagnostics-1592672-supplementary.pdf]

Table S1. CT imaging techniques used in the development and validation groups.

CT Techniques (N, %)	Total	Developmental Group	Validation Group
No. of patients	581	207	374
CT detector configuration (channels)			
16	105 (18.1%)	58 (28.0%)	47 (12.6%)
64	62 (10.7%)	21 (10.1%)	41 (11.0%)
128	414 (71.3%)	128 (61.8%)	286 (76.5%)
Tube voltage (kVp)			
100	573 (98.6%)	206 (99.5%)	367 (98.1%)
120	8 (1.4%)	1 (0.5%)	7 (1.9%)
Slice thickness			
3 mm	25 (4.3%)	3 (1.4%)	22 (5.9%)
5 mm	556 (95.7%)	204 (98.6%)	352 (94.1%)
CT vendors			
GE	7 (1.2%)	0 (0.0%)	7 (1.9%)
Siemens	574 (98.8%)	207 (100.0%)	367 (98.1%)

Data are shown as the number of patients and percentages.
